# Heart Rate Variability in Adolescents with Autistic Spectrum Disorder Practicing a Virtual Reality Using Two Different Interaction Devices (Concrete and Abstract): A Prospective Randomized Crossover Controlled Trial

**DOI:** 10.3390/healthcare13121402

**Published:** 2025-06-12

**Authors:** Étria Rodrigues, Ariane Livanos, Joyce A. L. Garbin, Susi M. S. Fernandes, Amanda O. Simcsik, Tânia B. Crocetta, Eduardo D. Dias, Carlos B. M. Monteiro, Fernando H. Magalhães, Alessandro H. N. Ré, Íbis A. P. Moraes, Talita D. Silva-Magalhães

**Affiliations:** 1 Medicine (Cardiology), Escola Paulista de Medicina, Federal University of São Paulo (EPM/UNIFESP), São Paulo 04021-001, SP, Brazil; etria.rodrigues@mackenzie.br (É.R.); joyce.garbin@unifesp.br (J.A.L.G.); ft.talitadias@gmail.com (T.D.S.-M.); 2 Department of Physiotherapy, Mackenzie Presbyterian University, São Paulo 01302-907, SP, Brazil; susifernandes@mackenzie.br; 3 Physical Activity Sciences, School of Arts, Science and Humanities, University of São Paulo (EACH-USP), São Paulo 03828-000, SP, Brazil; ariane.livanos@usp.br (A.L.); tania.crocetta@udesc.br (T.B.C.); carlosmonteiro@usp.br (C.B.M.M.); fhmagalhaes@usp.br (F.H.M.); alehnre@usp.br (A.H.N.R.); 4 Rehabilitation Sciences, Faculty of Medicine, University of São Paulo (FMUSP), São Paulo 01246-903, SP, Brazil; amanda.simcsik@usp.br (A.O.S.); edudati@gmail.com (E.D.D.); 5 Department of Physiotherapy, Federal University of Juiz de Fora Campus Governador Valadares (UFJF-GV), Governador Valadares 35020-360, MG, Brazil; 6 Graduate Program in Bioengineering, University Brazil, São Paulo 08230-030, SP, Brazil; 7 College of Medicine and Health, St Lukes Campus, University of Exeter, Exeter EX1 2LU, UK

**Keywords:** heart rate, autism spectrum disorder, virtual reality, autonomic nervous system

## Abstract

Individuals with autism spectrum disorder (ASD) often experience dysregulation of the autonomic nervous system, as evidenced by alterations in heart rate variability (HRV). It can be influenced by virtual reality (VR), which affects physiological responses due to its sensitivity to environmental and emotional stimuli. **Objectives:** This study aimed to assess HRV in individuals with ASD before, during, and after VR-based tasks over a 10-day period, specifically examining how HRV fluctuated in response to concrete (touchscreen) and abstract (webcam) interactions. **Methods:** Twenty-two male participants were randomly assigned to two sequences based on the order of tasks performed (starting with either the concrete or abstract task). **Results:** The findings revealed significant changes in HRV indices (RMSSD, SD1, SDNN, and SD2) between the two task types. **Conclusions:** The participants engaged in abstract tasks demonstrated higher motor demands, which were indicated by decreased parasympathetic activity and an increased LF/HF ratio, suggesting greater activation of the sympathetic nervous system.

## 1. Introduction

Autism spectrum disorder (ASD) is a complex neurodevelopmental condition marked by challenges in social communication and interaction, alongside the presence of repetitive behaviors and limited interests. It occurs more frequently in boys [1,2,3].

Individuals with ASD often present comorbidities, such as intellectual and language deficits, self-injurious behaviors, and specific motor difficulties, such as atypical gait and coordination problems [1,3]. The variability among individuals with ASD results in very distinct impacts and severity levels [2].

Although traditional therapies mainly focus on social and communication deficits, few interventions target the development of motor skills [4] and the effects resulting from motor activity practices. However, there has been a growing shift in therapeutic focus, directing attention toward improving motor skills, recognized as essential to promote greater autonomy and quality of life [5].

In the context of ASD, virtual reality (VR) has emerged as a promising area, as it offers a safe, repetitive, and adaptable platform for therapeutic interventions. This technology allows for the learning of motor tasks by individuals with ASD, with or without direct physical contact, broadening the possibilities for motor development [6,7,8]. However, the effect of prolonged VR exposure on heart rate variability (HRV) in individuals with ASD has not yet been investigated.

VR games provide an immersive experience that intensely activates the visual, auditory, and tactile senses [5,9]. This immersion can induce both physiological and psychological responses in the individual [10]. In the study by Moraes et al. [11], it was observed that VR tasks can be considered physical activities of very light intensity, in which heart rate increases were observed. Most participants also reported high enjoyment during the activities, suggesting that immersive experiences can influence motivation, positively affecting the VR experience, an important factor for engaging in physical activity [12].

Furthermore, the absence of physical restrictions in virtual environments allows individuals with ASD to explore and practice movements without fear of failure or injury, promoting a positive and encouraging learning experience. In a virtual environment, participants can perform motor tasks in a controlled and safe manner, with no real consequences for mistakes. This judgment-free environment encourages experimentation and risk-taking [13,14].

The integration of VR into therapies for individuals with ASD not only supports the acquisition of motor skills and their transfer to daily life but also opens new possibilities to explore the physiological effects of these activities [12]. Additionally, VR practice (abstract) has promoted improvements in motor skills in real tasks (concrete) across different populations [11,15].

Recent research suggests that VR may be effective in autonomic modulation, producing positive effects in different populations [7]. Studies show that adolescents with ASD may exhibit dysautonomia of the autonomic nervous system (ANS) [16]. Compared to typical developing adolescents, these individuals tend to display lower parasympathetic activity and higher sympathetic activity, suggesting a sympathetic-vagal imbalance and a reduction in HRV [17,18].

Autonomic cardiac regulation is an important health indicator, reflecting the ability of the ANS to adjust to the body’s demands. Variations in HRV patterns can be used as an early and effective diagnosis of physiological changes [19]. One of the most accessible, non-invasive, and reliable ways to identify autonomic dysfunctions, whether current or pre-existing, is the assessment of HRV through the fluctuations in the R-R intervals of heartbeats recorded by electrocardiograms [19]. However, as HRV has not yet been investigated in individuals with ASD during VR-based abstract tasks, its long-term effects on performance in real-world (i.e., concrete) activities remain unclear. This gap underscores the need for studies that examine the effectiveness of VR interventions in promoting changes in cardiac autonomic function.

Building on the previous description, we conducted a secondary analysis of the trial data (RBR-6c36pg), monitoring HRV in individuals with ASD as they performed a timing-coincident task during 10 days using two different devices: a touchscreen representing a concrete interface and a webcam representing an abstract interface.

The objective of this study was to observe HRV in individuals with ASD before, during, and after performing VR-based tasks. Specifically, we sought to assess how HRV fluctuates in response to both concrete (touchscreen) and abstract (webcam) interactions over a 10-day intervention period. Our hypothesis is based on evidence that HRV reflects the dynamic activity of the autonomic nervous system in response to physical and cognitive load [20]. Abstract tasks that involve more complex and varied upper limb movements, such as those captured by webcam interactions, demand greater sensorimotor coordination and attentional resources [15], which may lead to an alteration of HRV indices. Furthermore, repeated engagement in moderate-intensity physical or cognitive activities can promote longitudinal adaptation [11], reflected in increased HRV over time [21].

## 2. Materials and Methods

### 2.1. Trial Design

This report represents a secondary arm of the longitudinal, retrospective, randomized crossover controlled trial conducted by Moraes et al. [11], which investigated the use of VR activities to assess heart rate variability. The study received approval from the Research Ethics Committee of the Federal University of São Paulo, CAAE: 50229521.2.0000.5505, and was registered in the Brazilian Clinical Trials Registry under the identification number RBR-6c36pg (date of registration: 18 June 2018).

The intervention phase of the study took place between July 2018 and July 2019 at the Integrated Psycho-Pedagogical Support Group (GAPI)—Special Education School, located in São Bernardo do Campo, São Paulo, Brazil, an institution specializing in the care of children and adolescents with developmental disorders. The study was conducted in accordance with the guidelines and recommendations of the Consolidated Standards of Reporting Trials (CONSORT) 2010 statement [22,23].

### 2.2. Participants

Much of the methodology employed in this secondary analysis mirrored that described in the report by Moraes et al. [11]. The study included volunteers between 10 and 16 years of age, all diagnosed with ASD by a neurologist and a multidisciplinary team. The diagnosis was established through a comprehensive evaluation of neurodevelopmental history, complemented by psychological, communication, and psychiatric assessments.

All participants enrolled in the study signed a Free and Informed Assent form, while their guardians completed the Free and Informed Consent Form; both documents had been previously approved by the Research Ethics Committee. All data from the study are publicly available [24].

### 2.3. Inclusion and Exclusion Criteria

Participants were included if they met the following criteria: (1) the provision of signed free and informed consent by parents or guardians and free and informed assent by the participant; (2) male adolescents; (3) diagnosis of mild or moderate ASD with sufficient cognitive ability to comprehend and perform the proposed tasks; (4) no use of medications capable of affecting the study variables, such as beta-blockers; and (5) the absence of comorbidities, including attention deficit hyperactivity disorder and Down syndrome.

Exclusion criteria were: (1) the inability to perform the task correctly after three guided attempts, with explanations and demonstrations by the evaluator, resulting in one exclusion on the first day, and (2) withdrawal from the study at any stage, which led to one exclusion on the fifth day.

### 2.4. Sample Characterization

An initial assessment of the pre-selected participants was carried out two days before the start of the intervention protocol (D0), utilizing the Wechsler Intelligence Scale (WISC), the Childhood Autism Rating Scale (CARS), and the Pediatric Evaluation of Disability Inventory (PEDI).

#### 2.4.1. Wechsler Intelligence Scale

The Intelligence Quotient (IQ) is commonly used as an indicator of global cognitive functioning and serves as a structural measure in case-control studies of neurodevelopmental disorders [25]. In this study, the abbreviated version of the WISC-III was administered by a psychologist from the GAPI institution [26]. IQ scores are classified as follows: mild intellectual disability with a score ranging from 55 to 70, borderline intelligence between 70 and 85, normal intelligence at 85 or above, above-average intelligence between 115 and 129, and superior intelligence at 130 or higher [27].

#### 2.4.2. Childhood Autism Rating Scale

The CARS was created to diagnose and evaluate the severity of autism. It includes 15 behavioral items, assessed in conjunction with clinical judgment, covering various domains, such as interpersonal relationships, imitation, emotional responses, body usage, object use, reaction to changes, visual and auditory responses, responses to and use of taste, smell, and touch, fear or anxiety, verbal and nonverbal communication, activity level, consistency of intellectual response, and overall impressions [28,29].

Each item on the CARS is rated on a scale from 1 (no pathology) to 4 (severe pathology), with total scores computed as “raw scores”. The severity of autism is classified as follows: a score between 15 and 29 indicates non-autistic, 30 to 36 indicates mild to moderate autism, and 37 to 60 indicates severe autism [28,29].

#### 2.4.3. Pediatric Evaluation of Disability Inventory

The PEDI is a questionnaire conducted through interviews with caregivers, clinical judgment from therapists or educators working with the child, or through direct observation of the child performing tasks [30,31]. It offers a comprehensive description of the child’s functional abilities, documenting both capacity and performance across two primary domains: (1) functional skills and (2) caregiver assistance. These domains are further subdivided into three areas: (1) self-care, (2) mobility, and (3) social function [30,31].

### 2.5. Sample Size

The sample size for the primary outcome measure was calculated using G*Power 3.1.5 statistical software, based on data from five patients in a pilot study. With a power of 0.80, an alpha level of 0.05, and an effect size of 0.65 (Cohen’s d), the calculation indicated that 20 participants (10 per group) would be required. To compensate for a possible 20% dropout rate, the study included 24 participants.

### 2.6. Abstract Activity

The researchers responsible for implementing the intervention protocol were not involved in the recruitment, initial evaluation, or randomization stages. MoveHero software, version 2 (2017), was used for the VR tasks, which took place individually in a designated room equipped with a computer, table, and chair, under the supervision of a qualified evaluator. Participants were seated comfortably in chairs adjusted to their size and needs. Prior to starting the task, the examiner provided a verbal explanation and demonstration of how to play the VR game. To ensure comprehension, participants completed a single task session on the platform before beginning the actual task.

**Figure 1:**
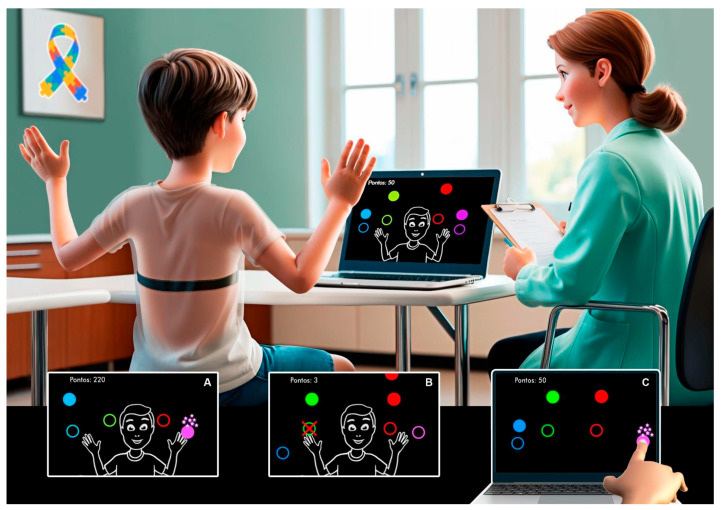
Representative design of a participant engaging with the MoveHero software task (Research Group and Technological Application in Rehabilitation—PATER Group, São Paulo, Brazil). (**A**) Illustration showing a successful hit by the participant, with the targeted sphere highlighted. (**B**) Illustration depicting a miss by the participant, indicated by a red X on the sphere. (**C**) Illustration of the task being performed using the touchscreen. The spheres are ordered from left to right in the following colors: blue, green, red, and purple.

The game, developed by the School of Arts, Sciences, and Humanities at the University of São Paulo (EACH-USP), involves spheres that fall into four imaginary columns on the computer screen, accompanied by a song chosen by the researcher in accordance with the software’s guidelines [32]. Participants are required to interact with the spheres as they reach their designated targets—four in total—each corresponding to the sphere’s color. These targets are positioned in parallel at two different heights, with two on the left and two on the right (Figure 1).

The VR game tracks the participant’s movements using a webcam, enabling interaction without physical contact. Participants move their arms about one meter away from the computer screen to align with the targets displayed and interact with the falling spheres. Alternatively, they can use the touchscreen for more direct interaction, which requires physical contact with the screen. To interact successfully, participants must wait for the spheres to align with one of the target circles. The game provides immediate feedback, displaying a glowing signal above the target when an interaction is successful. Additionally, the total score is shown in the lower-left corner of the screen, with each successful hit earning 10 points.

### 2.7. Heart Rate Variability

All heart rate recordings were obtained using a heart rate monitor. A recording strap was placed across the participant’s chest, while a Polar V800 HR receiver (Polar Electro, Kempele, Finland) was positioned nearby. Participants sat comfortably in a standard chair, with their hands resting on their legs and feet flat on the floor. Heart rate variability (HRV) indices were calculated by analyzing the intervals between R waves recorded by the monitor. These data were then transferred to the Polar ProTrainer software (version 3.0, Polar Electro, Kempele, Finland) for heart rate visualization and cardiac period extraction in ‘.txt’ file format.

HRV data were collected in three phases: (1) Heart rate variability (HRV) data were collected two days before the start of the intervention, with participants seated quietly at rest for 15 min; (2) for the intervention data, both real and virtual activities were performed and recorded for 12 min over a span of 10 days; (3) finally, two days after the conclusion of the activities, resting HRV data were collected again.

All recorded data were transferred to the software for storage, filtering, and further analysis. Heart rate variability (HRV) was analyzed using Kubios HRV^®^ software (version 1.1 for Windows, Biomedical Signal Analysis Group, Department of Applied Physics, University of Kuopio, Kuopio, Finland). The extracted indices were assessed using linear methods in both the time and frequency domains, along with Poincaré plot analysis [19].

First, in the time domain analysis, the following indices were obtained: (1) mean HR, representing the average of all normal heartbeats, expressed in beats per minute (bpm); (2) mean RR, the mean of all normal RR intervals, also expressed in bpm; (3) standard deviation of normal RR intervals (SDNN), reflecting the standard deviation of all normal RR intervals over a given period, expressed in milliseconds. The SDNN, derived from long-term recordings, provides an indication of overall autonomic modulation but does not distinguish between increased sympathetic activity or decreased parasympathetic tone [19]; (4) root mean square of successive differences (RMSSD), which measures the square root of the mean squared differences between successive normal RR intervals, expressed in milliseconds, and specifically reflects parasympathetic activity [19]; and (5) percentage of normal RR intervals differing by more than 50 ms (pNN50), indicating the proportion of successive RR intervals that differ by more than 50 ms, also reflecting parasympathetic modulation [19].

Next, in the frequency domain analysis, the following indices were evaluated: (1) low frequency (LF), which reflects the combined influence of vagal and sympathetic activity on the heart, with a predominance of sympathetic modulation, expressed in normalized units [19]; (2) high frequency (HF), which represents respiratory-related vagal activity affecting the heart, also expressed in normalized units [24]; and (3) the LF/HF ratio, which captures both absolute and relative shifts between sympathetic and parasympathetic components of the autonomic nervous system (ANS), providing an indication of the balance between sympathetic and vagal influences [19].

In the Poincaré plot analysis, the following indices were used: (1) SD1, which represents the instantaneous beat-to-beat variability and reflects parasympathetic activity [17]; and (2) SD2, which indicates heart rate variability in long-term recordings and reflects global variability [19].

### 2.8. Randomization

At baseline (D0), volunteers were evaluated and included in the protocol according to the established inclusion criteria. Participants and their guardians were then presented with the consent and assent forms, along with a detailed explanation of the study’s procedures and objectives. After signing the forms, data were collected to characterize the sample. Following this, adolescents were randomly assigned in a 1:1 ratio to two sequences (A and B) using the website randomization.com. The randomization process was carried out by an independent researcher who was not involved in participant recruitment or assessments, ensuring a blinded allocation.

Next, the initial HRV assessment was performed while participants remained seated at rest, during which HRV data were recorded. Following this, the intervention phase began using the MoveHero virtual game, which had previously been explained and demonstrated by the researcher. The intervention consisted of 10 sessions, conducted twice weekly for 12 min per session, with each participant practicing on each interface across five sessions. In total, the intervention spanned six weeks.

The intervention sequences were organized as follows:

Sequence A: Participants first engaged with the abstract interaction via the webcam interface, followed by the concrete task using the touchscreen interface.

Sequence B: Participants first completed the concrete interaction using the touchscreen interface, followed by the abstract task utilizing the webcam interface.

At the end of the intervention period, participants’ HRV was reassessed at rest, following the same procedures as the initial evaluation.

### 2.9. Flow OS Participants

**Figure 2:**
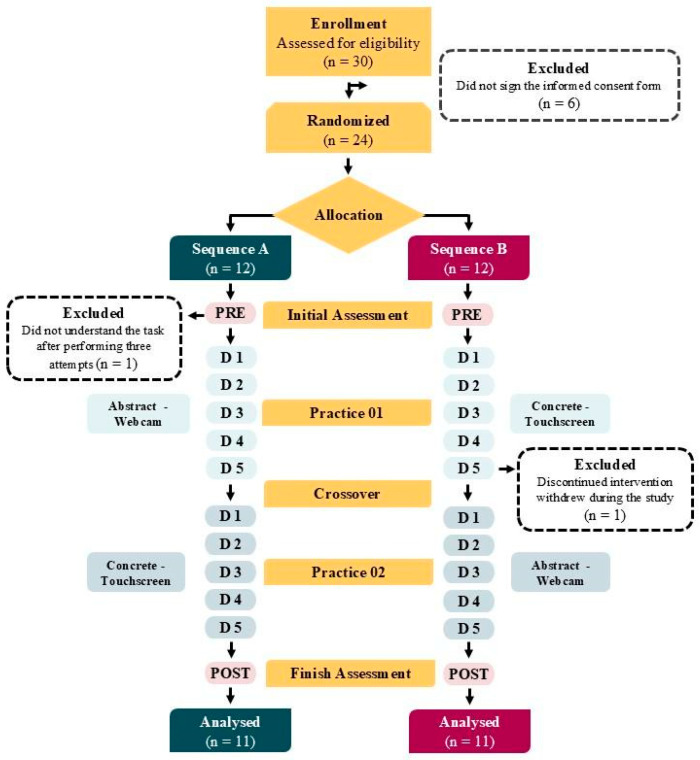
Study design.

As shown in Figure 2, 30 patients were assessed for eligibility; however, 24 participants agreed to participate and were randomized into two sequences. Sequence A completed the abstract task (webcam) for five days (Practice 1), followed by the concrete task (touchscreen) after the crossover (Practice 2). Sequence B followed the reverse order. HRV was measured before the intervention (Pre/Rest), throughout all practice days, and again after the second practice block (Post). Two participants, one from each group, were excluded. In total, 22 participants completed the full protocol and were included in the final analysis.

### 2.10. Data Analysis

For the analysis of independent variables, the chi-square test was applied to categorical variables, while continuous variables were examined using Student’s *t*-test. As dependent variables, we considered the heart rate variability (HRV) indices, including both time and frequency domain measures. To analyze comparisons between baseline (Pre) and final assessments (Post), data were subjected to a MANOVA, structured as 2 (Sequences: A—abstract-concrete and B—concrete-abstract) by 2 (Assessments: pre vs. post; pre vs. day 5; or pre vs. day 10), with repeated measures on the second factor. For the comparisons across activities, another MANOVA was conducted with 2 (Type of Intervention: Abstract vs. Concrete) by 2 (Sequences: A vs. B) by 5 (Days of intervention for each activity), with repeated measures on the last two factors. Post-hoc analyses were performed using the Least Significant Difference (LSD) test.

The data are presented as mean values with standard error. The effect size was quantified using partial Eta squared (η_p_^2^), categorized as small (effect size > 0.01), medium (effect size > 0.06), or large (effect size > 0.14). Additionally, observed power (op) was reported. To assess the differences between the two interfaces (Concrete vs. Abstract) while disregarding the practice sequence, an ANOVA was performed to check for significant differences between them.

Furthermore, a regression analysis was conducted to evaluate whether the independent variables (age, body mass index, IQ, CARS, and PEDI) had an impact on changes in HRV indices. The dependent variable in this analysis was the difference in the three indices (SDNN, RMSSD, LF/HF) between rest post-assessment and rest pre-assessment for both sequences. All statistical analyses were performed using SPSS version 26.0, with *p*-values < 0.05 considered significant. Tables containing all mean values, as well as the F values, *p*-values, eta squared, and observed power for the main analyses, are provided in the Supporting Materials (Tables S1 and S2).

## 3. Results

A total of 30 individuals with ASD were initially invited to participate in the study. Of these, 6 did not provide consent, leaving 24 male participants who were included in the protocol. One participant was later excluded due to a lack of understanding of the task, and another withdrew during the study, not completing the required training days. This resulted in a final sample of 22 participants, who were randomly assigned to two Sequences based on the order in which they performed the tasks (starting with either the concrete or abstract task).

All participants reported prior experience with commercial non-immersive VR games. Table 1 presents the mean values and their dispersion for the independent variables. Given the heterogeneity of individuals with ASD, scales assessing intellectual functioning, autism severity, and functional abilities were used to ensure a more comprehensive understanding of the sample. No statistically significant differences were found between groups for these variables, confirming that the Sequence groups were homogeneous.

### 3.1. Heart Rate Variability Measurements

**Figure 3:**
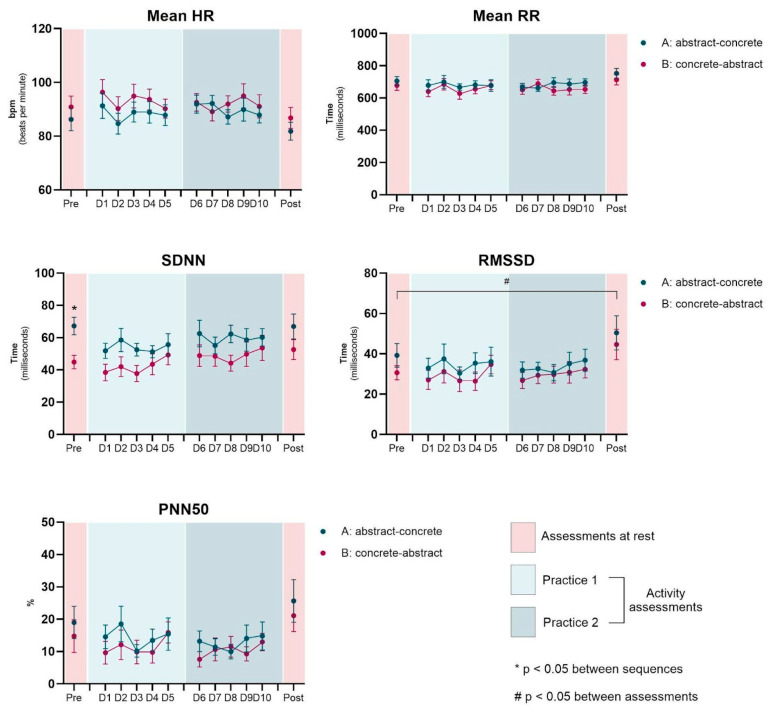
Representation of the mean and standard error of HRV indices (Time domain: Mean HR, Mean RR, SDNN, RMSSD, and PNN50) across sequences (A: abstract-concrete and B: concrete-abstract) and assessments (Rest–pre and post, Activity—D1 to D10).

**Figure 4:**
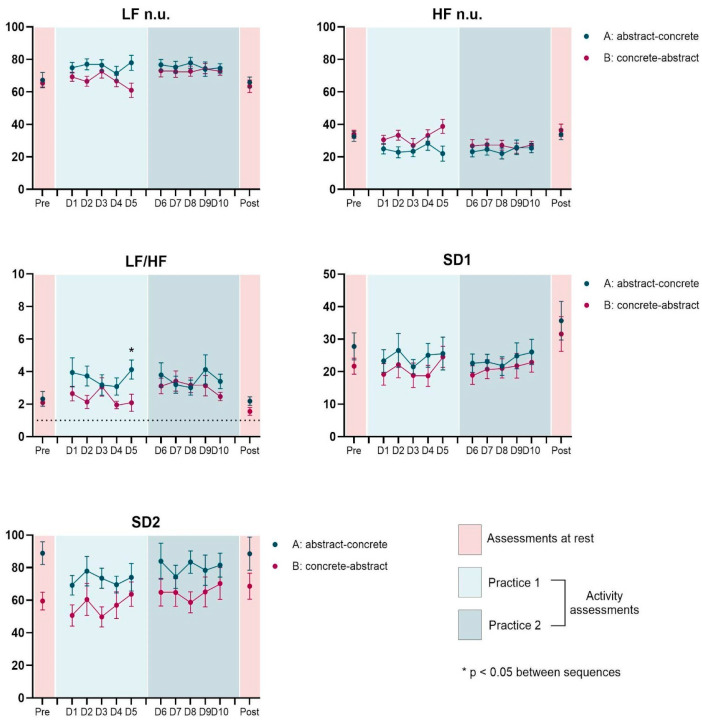
Representation of the mean and standard error of HRV indices (Frequency domain: LF n.u., HF n.u. and LF/HF; and Poincaré plot analysis: SD1 and SD2) across sequences (A: abstract-concrete and B: concrete-abstract) and assessments (Rest–pre and post, Activity—D1 to D10). In the LF/HF graph, the dotted line indicates the ideal reference value of 1 for this measure.

MANOVA did not show any significant effects, and no interactions were found between the factors. Separate repeated measures ANOVAs (RM-ANOVAs) for each HRV index are detailed in the following paragraphs (Table 2 and Figure 3 and Figure 4).

### 3.2. Pre vs. Post Assessments

The ANOVA revealed significant main effects for Moments for RMSSD (F_1, 20_ = 5.77; *p* = 0.026, η_p_^2^ = 0.22; op = 0.63) and SD1 (F_1, 20_ = 5.78; *p* = 0.026, η_p_^2^ = 0.22; op = 0.63), and a marginally significant effect, but with a large effect size for Mean RR (F_1, 20_ = 4.06; *p* = 0.058, η_p_^2^ = 0.17; op = 0.48), which shows an increase in RMSSD and SD1 at rest from pre (34.9 ms and 24.7, respectively) to post intervention (47.5 ms and 33.6) in both sequences.

### 3.3. Effect of Interventions (Rest-Pre to Activity on D1)

ANOVA showed that there were significant main effects for SDNN (F_1, 20_ = 11.9; *p* = 0.003, η_p_^2^ = 0.37; op = 0.91) and SD2 (F_1, 20_ = 10.7; *p* = 0.004, η_p_^2^ = 0.35; op = 0.88). Post-hoc comparisons indicated that only the group engaged in the abstract environment activity showed a decrease in SDNN, HF n.u and SD2 (Figure 3 and Figure 4), and an increased LF/HF ratio from rest to activity (SDNN: 67.2 to 51.8 ms, *p* = 0.003; HF n.u.: 32.6 to 24.9, *p* = 0.048; SD2: 88.9 to 69.2, *p* = 0.005; LF/HF ratio: 2.319 to 3.950, *p* = 0.049).

### 3.4. Effect of Interventions (Concrete vs. Abstract)

ANOVA revealed a marginally significant main effect, but with high effect size for the LF/HF ratio (F_1, 20_ = 3.63; *p* = 0.071, η_p_^2^ = 0.15; op = 0.44), with a mean of 2.94 for Concrete intervention and 3.34 for Abstract intervention. No significant effects were found for the other indices (Figure 3 and Figure 4).

### 3.5. Effect of Practice (D1 × D5)

No significant main effects were observed during practice; however, post-hoc tests revealed that the group practicing in the Concrete environment during the first sequence showed an increase in SDNN (M = 38.4 to 49.3, *p* = 0.082) and HF n.u. (M = 30.5 to 38.8, *p* = 0.079), as well as a decrease in LF n.u. (M = 69.3 to 61.0, *p* = 0.079) from D1 to D5.

### 3.6. Effect of Sequence (Concrete First vs. Abstract First)

The post hoc test showed significant effects for SDNN (*p* = 0.046), LF n.u. (*p* = 0.027), HF n.u. (*p* = 0.026), LF/HF ratio (*p* = 0.031) and marginally for SD2 (*p* = 0.070), showing that the group that performed in the Concrete environment first presented a significant increase in SDNN, LF n.u., LF/HF ratio and SD2 from Concrete (42.2 ms, 67.3, 2.38, 56.3, respectively) to Abstract environment (49.0 ms, 73.1, 3.1, 64.7, respectively) and a significant decrease in HF n.u. (Concrete: 32.5, Abstract: 26.8). The group that performed the intervention in the Virtual environment first did not show significant changes when performed further in the Real environment (Figure 3 and Figure 4).

### 3.7. Regression Analysis

The regression analysis showed no significant effects, suggesting that the independent variables, including intellectual functioning, autism severity, and functional abilities, had no influence on the variations observed in HRV indices (SDNN, RMSSD, and LF/HF).

## 4. Discussion

In this study, we investigated the effects of concrete and abstract VR tasks on the ANS in adolescents with ASD, focusing on HRV as a measure of autonomic regulation. Based on the physical demands of the VR tasks, we hypothesized that engaging in both types of activities would result in distinct HRV patterns, with an overall increase in HRV as the ANS adapted to repeated practice. Specifically, we anticipated that the abstract tasks, which require a broader range of movements, would present more autonomic responses to the demand of the activity compared to the concrete tasks. Our findings partially confirmed this hypothesis, revealing significant changes in HRV in some indices that underscore the potential of VR as a therapeutic tool for improving autonomic function and cardiovascular health in individuals with ASD, and those results will be discussed as follows.

### 4.1. Improvements in HRV Indices After 10 Days of Intervention

Our study revealed that a 10-day VR-based intervention resulted in significant improvements in HRV indices at rest among adolescents with ASD. The rise in RMSSD and SD1 values from pre to post interventions in both sequences, which reflect parasympathetic activity, further supports the beneficial chronic effects of the intervention on autonomic regulation. These findings align with previous research suggesting that regular physical activity can positively influence cardiovascular health by promoting a shift towards greater parasympathetic activity at rest [33].

Given that individuals with ASD typically exhibit lower HRV due to autonomic dysregulation [34,35], our results highlight the potential of VR-based interventions to counteract these deficits and promote cardiovascular health. This is particularly crucial for this population, as reduced HRV, particularly with lower parasympathetic modulation at rest, is associated with an increased risk of cardiovascular conditions such as hypertension and myocardial infarction [19]. Therefore, VR interventions can be considered as a tool for the promotion of physical activity that offers cardioprotective benefits, emphasizing the importance of regular physical activity for individuals with ASD [36].

These improvements were observed in both sequences. Specifically, we noted an increase in RMSSD and SD1 values, indicating enhanced HRV, which is a marker of improved autonomic function. One possible explanation for the increase in RMSSD and SD1 values in both groups could be that the repeated practice of VR tasks, regardless of whether they involved concrete or abstract interactions, led to a general improvement in autonomic regulation. This could be due to the engaging and movement necessity to complete the VR task proposed, which may stimulate both cognitive and motor systems, promoting a balanced activation of the autonomic nervous system [37,38]. The consistency of this engagement over the 10-day intervention period may have fostered adaptations that enhanced HRV.

Studies suggest that VR experiences that require physical engagement can enhance HRV by promoting parasympathetic activity, which is often linked to relaxation and improved autonomic balance [39]. The continuous interaction and movement demanded by VR tasks (abstract) might contribute to these autonomic improvements, as these abstract tasks have been shown to elicit physiological responses similar to real-life experiences, thus potentially fostering adaptations that enhance HRV [40].

### 4.2. Rest vs. First Day of Activity: Higher LF/HF Ratio in Abstract Tasks

When comparing HRV indices between the initial rest period and the first day of VR activities, significant changes were observed. The decrease in the HF index from rest to activity shows that there is a vagal withdrawal only during abstract tasks, and the decrease in SDNN and SD2 and the increase in the LF/HF ratio corroborate the higher sympathetic demand, as the parasympathetic modulation decreased, as seen in scenarios that require cognitive or physical effort [41].

In the sequence that began with the VR task in a concrete environment, the anticipated autonomic response did not occur, suggesting that this environment did not demand significant autonomic engagement. The lack of significant autonomic response during the concrete VR task might be due to its less demanding nature, as certain VR environments do not always elicit strong autonomic shifts unless they involve intense or abstract challenges. This aligns with studies showing that VR tasks with minimal cognitive load do not evoke the same autonomic shifts as more abstract or challenging environments [42,43,44].

In abstract tasks, the degree of task complexity influences the extent of autonomic modulation; the demanding tasks tend to induce higher cognitive loads. By contrast, environments that are more concrete and less mentally taxing may not sufficiently stimulate these autonomic shifts, as observed in research where high cognitive load led to significant changes in ANS markers like HRV compared to low-load tasks [42,43]. These findings reinforce the idea that VR tasks with minimal cognitive demands may not evoke the same level of autonomic adjustment as more complex or abstract VR activities, which require heightened cognitive and emotional engagement.

### 4.3. Differences Between Concrete and Abstract Tasks

When analyzing purely concrete versus abstract tasks, disregarding the sequences (item 4.3), our results show that the abstract task promotes a higher sympathetic demand, as the LF/HF ratio was higher in this group [41,42,43,44,45]. Also, the effect of the practice during 5 days of intervention in each task (item 4.4) shows that the group that practiced in the concrete environment in the first sequence increased parasympathetic modulation (HF index) throughout the days of practice, while the group that performed the first practice on the abstract task did not present significant differences throughout the first 5 days.

Finally, when analyzing the effect of the sequence, i.e., whether there was or was not a significant difference when changing from concrete to abstract environments after 5 days of practice and vice versa (item 4.5), it was possible to see that the group that performed the first sequence in the concrete environment significantly increased HRV when performing the second sequence in the abstract environment by increasing the SDNN, LF n.u., the LF/HF ratio, and SD2, and this is also due to the lower parasympathetic modulation during the abstract task when compared to the concrete task, as shown by the decrease in HF n.u. Conversely, those who began with abstract tasks did not exhibit significant changes when switching to concrete interaction.

These findings suggest that the order of task exposure may influence autonomic responses, potentially due to initial adaptations that prepare the ANS for subsequent challenges. This versatility makes VR a valuable tool in therapeutic settings, as it can be tailored to various cognitive and motor demands without being constrained by the type of interface used. These results reinforce the idea that VR-based interventions can offer flexible and effective rehabilitation options, catering to the unique needs of individuals with ASD [10,37].

When comparing HRV indices between the initial rest period and the first day of VR activities, significant changes were observed, particularly in the LF/HF ratio, which increased during the abstract (webcam) tasks. This increase suggests a shift towards greater sympathetic activation during these tasks, reflecting their higher cognitive and motor demands compared to the concrete (touchscreen) tasks. Both sequences (A and B) showed an increase in sympathetic activity and a decrease in global variability (SDNN) during the VR tasks, consistent with the physiological response to physical exertion [46]. These results suggest that abstract tasks in VR, which require more complex and broader motor movements, place a greater demand on the ANS, leading to a more pronounced sympathetic response. This finding underscores the utility of VR as a tool for inducing physiological changes akin to those observed during traditional physical activity, further validating its use in rehabilitation for individuals with ASD.

### 4.4. VR Tasks to ASD Population Benefits

According to Healy et al. [36], physical activity for individuals with ASD can generate positive effects for this population that include the development of muscular strength and endurance, physical conditioning, locomotor skills, manipulative skills, and social skills. Health problems such as systemic arterial hypertension, acute myocardial infarction, coronary insufficiency, and atherosclerosis may be related to reduced HRV indices [19]. Studies such as Sandercock, Bromley, and Brodie [47] demonstrate that physical activity can improve global HRV in certain populations.

Research indicates that individuals with ASD tend to have lower HRV due to atypical functioning of the ANS, which often involves reduced values in mean RR and RMSSD, and an altered LF/HF ratio. Specifically, studies have shown that children with ASD frequently display lower overall HRV, reflecting diminished parasympathetic activity and increased sympathetic dominance. This reduced parasympathetic modulation is often associated with challenges in autonomic regulation and may impact responses to stress and environmental stimuli. In particular, individuals with ASD tend to show abnormalities in HRV indices like RMSSD, which reflects vagal tone and is typically lower in this population, signifying decreased parasympathetic influence. Additionally, an elevated LF/HF ratio in individuals with ASD suggests a sympathetic predominance, which is often interpreted as a sign of autonomic imbalance.

It is important to note that most existing findings on HRV in individuals with ASD have been derived from adult samples. For instance, Thapa et al. [35] reported reduced HRV in adults with ASD, including lower RMSSD and other time- and frequency-domain indices. These results are consistent with broader evidence of autonomic dysregulation in ASD across several studies [35,48,49]. However, findings based on adult populations may not fully capture the developmental patterns observed in children and adolescents. Younger individuals with ASD may exhibit distinct autonomic profiles due to ongoing neurodevelopmental processes. In typically developing populations, HRV tends to increase with age, reflecting vagal maturation and enhanced autonomic flexibility [50]. In contrast, this developmental trajectory may be disrupted in individuals with ASD, leading to atypical patterns of autonomic regulation during childhood and adolescence [35,51]. These changes were studied by Kalfiÿrt et al. [34], who evaluated children with ASD and children with typical development. Their evaluations found higher SDNN values (corresponding to global variability) in children with typical development. These results corroborate those found by Thapa et al. [35], who found, in the HF and RMSSD measurements (both represent parasympathetic activity), a reduction in HRV in children diagnosed with ASD.

This study presents some limitations that should be considered when interpreting the findings. Although MANOVA was appropriate for the structure and objectives of our analysis, alternative statistical approaches such as linear mixed-effects models could offer advantages in future studies, particularly in accounting for individual variability and potential data imbalances. Additionally, while our findings are consistent with broader evidence of autonomic dysregulation in ASD, they are most applicable to pediatric populations and may not generalize directly to adults. Future research should investigate age-specific trajectories of HRV in individuals with ASD to clarify how developmental processes influence autonomic function over time. The VR tasks used in this study differed in their interface modalities but were not standardized or objectively quantified in terms of cognitive or physical load, which limits the precision in attributing HRV changes to specific task demands. Other potentially influential variables, such as physical activity level, sleep quality, and sensory profiles, were not controlled. Finally, the relatively small and homogeneous sample limits the generalizability of the findings. The absence of female participants and a broader developmental range restricts the applicability of these results across the full autism spectrum. Larger, more diverse samples are necessary to strengthen external validity and to explore the potential moderating effects of gender, age, and comorbidities on autonomic function in ASD.

## 5. Conclusions

Individuals with ASD who underwent a 10-day VR intervention involving both concrete (touchscreen) and abstract (webcam) tasks showed significant changes in HRV indices, particularly RMSSD, SD1, SDNN, and SD2, with distinct patterns between the two task types. Participants performing abstract tasks demonstrated higher motor demands, reflected in decreased parasympathetic activity and an increased LF/HF ratio, indicating greater sympathetic nervous system activation.

## Figures and Tables

**Table 1:** Comparison of the independent variables between Sequence A (abstract-concrete) and Sequence B (concrete-abstract).

Variable	Sequence A		Sequence B		*p*-Value
	Mean ± SD	CI [LL, UL]	Mean ± SD	CI [LL, UL]	
Age (years)	14.1 ± 1.7	[12, 15]	13.9 ± 1.9	[12, 15]	0.822
Height (meters)	1.65 ± 0.13	[1.57, 1.73]	1.67 ± 0.11	[1.59, 1.75]	0.693
Weight (kilograms)	61.9 ± 14.8	[51, 72]	63.0 ± 18.8	[52, 73]	0.877
BMI (kg/m^2^)	22.0 ± 4.0	[19, 25]	22.0 ± 6.0	[19, 25]	0.899
IQ	87.6 ± 11.3	[79, 95]	83.13 ± 13.6	[75, 91]	0.412
CARS	34.3 ± 1.3	[33, 35]	33.7 ± 1.7	[32, 34]	0.348
PEDI—FS Self-Care	89.6 ± 11.3	[82, 96]	91.8 ± 10.1	[85, 98]	0.648
PEDI—FS Mobility	66.3 ± 7.7	[62, 69]	67.7 ± 1.6	[64, 71]	0.586
PEDI—FS Social Function	77.5 ± 9.8	[71, 83]	77.8 ± 9.9	[71, 84]	0.947
PEDI—CA Self-Care	94.2 ± 9.0	[89, 99]	97.0 ± 6.7	[92, 102]	0.419
PEDI—CA Mobility	92.4 ± 15.0	[85, 99]	96.8 ± 5.7	[89, 103]	0.377
PEDI—CA Social Function	88.0 ± 11.5	[80, 95]	87.1 ± 12.8	[79, 94]	0.867

Legend: BMI: Body Mass Index; IQ: Intelligence Quotient; CARS: Childhood Autism Rating Scale; SD: Standard Deviation; CI: Confidence Interval; LL: Lower Limit; UL: Upper Limit; PEDI: Pediatric Disability Assessment Inventory; FS: Functional Skills; CA: Caregiver Assistance.

**Table 2:** Descriptive statistics of the main HRV measures and significance values (*p*) for main effects in the MANOVA.

Variable	S	Pre-Rest		Day 1—D1		Day 5—D5		Day 10—D10		Post		*p* (Pre vs. Post)			*p* (Pre vs. D1)			*p* (Pre vs. D10)			*p* (D1 vs. D5)		
		Mean	SD	Mean	SD	Mean	SD	Mean	SD	Mean	SD	M	M × S	S	M	M × S	S	M	M × S	S	M	M × S	S
Mean RR	A	705.5	91.8	667.2	76.0	696.3	75.7	677.2	117.8	753.1	101.8	0.058	-	-	*-*	-	-	-	-	-	-	-	-
	B	678.2	101.5	640.6	109.5	679.0	89.6	653.7	85.6	713.1	104.6												
Mean HR	A	86.2	14.0	91.9	11.1	87.9	9.8	87.8	12.8	81.8	11.0	-	-	-	-	-	-	-	-	-	-	-	-
	B	90.9	13.5	96.4	15.4	90.2	11.6	91.1	14.3	86.8	12.9												
SDNN	A	67.3	18.2	62.6	27.3	60.2	18.1	55.7	22.4	66.9	25.6	-	-	0.020	-	0.024	-	-	0.039	-	-	-	-
	B	44.9	13.8	38.4	16.9	49.3	20.3	53.7	25.9	52.6	20.5												
RMSSD	A	39.2	19.5	31.9	13.3	36.8	18.0	36.1	23.7	50.4	27.9	0.026	-	-	-	-	-	-	-	-	-	-	-
	B	30.6	11.6	27.1	15.5	34.7	15.3	32.3	14.2	44.6	24.9												
pNN50	A	19.0	16.5	13.2	10.5	14.8	14.3	15.4	16.6	25.7	21.8	-	-	-	-	-	-	-	-	-	-	-	-
	B	14.8	16.7	9.6	11.6	15.9	10.8	13.0	8.9	21.1	16.3												
LF n.u.	A	67.2	15.7	76.7	10.7	74.5	9.6	77.9	15.3	65.9	10.7	-	-	-	-	0.014	-	0.001	-	-	-	-	-
	B	65.4	8.1	69.3	9.2	61.0	14.5	72.8	8.1	63.5	12.8												
HF n.u.	A	32.6	10.3	23.2	10.7	25.5	9.6	22.0	15.3	33.8	10.6	-	-	-	-	0.022	0.046	0.005	-	-	-	-	-
	B	33.9	8.1	30.5	9.2	38.8	14.4	27.1	8.1	36.4	12.7												
LF/HF	A	2.3	1.5	3.8	2.5	3.4	1.4	4.1	1.9	2.2	0.9	-	-	-	0.010	0.009	-	<0.001	0.011	-	-	-	-
	B	2.1	0.7	2.7	1.5	2.1	1.7	2.5	0.8	1.6	0.8												
SD1	A	27.8	13.8	22.6	9.4	26.1	12.8	25.6	16.8	35.7	19.8	0.026	-	-	-	-	-	-	-	-	-	-	-
	B	21.7	8.2	19.2	11.0	24.6	10.8	22.9	10.0	31.6	17.6												
SD2	A	88.9	23.1	84.0	36.9	81.5	24.3	74.1	28.0	88.6	33.6	-	-	0.017	-	0.030	0.045	-	0.043	-	-	-	-
	B	59.5	18.1	50.7	21.8	63.7	24.7	70.3	32.4	68.6	26.5												

Legend: A: Participants first engaged with the abstract interaction via the webcam interface, followed by the concrete task using the touchscreen interface; B: Participants first completed the concrete interaction using the touchscreen interface, followed by the abstract task utilizing the webcam interface; M: Moments of assessments (Pre, D1 to D10, and Post); S: Sequence.

## Data Availability

The original data presented in the study are openly available in Mendeley Data [29].

## Supplementary Materials

The following supporting information can be downloaded at: https://www.mdpi.com/article/10.3390/healthcare13121402/s1, Table S1: All mean values from the assessment time points—Moments (Pre, D1 to D10, and Post) for both sequences A and B; Table S2: Summary of MANOVA Results: F Values, *p*-Values, Eta partial square (η_p_^2^), and Observed Power for the Main Analyses. Only data with statistical significance were reported.

## Abbreviations

ANS	Autonomic Nervous System
ASD	Autism Spectrum Disorder
BPM	Beats Per Minute
CARS	Childhood Autism Rating Scale
GAPI	Integrated Psychopedagogical Support Group
HF	High Frequency
HRV	Heart Rate Variability
IQ	Intelligence Quotient
LF	Low Frequency
LSD	Least Significant Difference
PEDI	Pediatric Evaluation of Disability Inventory
pNN50	Percentage of Normal RR Intervals Differing by More Than 50 ms
RMSSD	Root Mean Square of Successive Differences
RR	Interval between R waves of the PQRST complex
SDNN	Standard Deviation of Normal RR Intervals
VR	Virtual Reality
WISC	Wechsler Intelligence Scale
