# Supplementary material for: Heart Rate Variability in Adolescents with Autistic Spectrum Disorder Practicing a Virtual Reality Using Two Different Interaction Devices (Concrete and Abstract): A Prospective Randomized Crossover Controlled Trial
Source: Healthcare (Basel). 2025 Jun 12;13(12):1402. doi: 10.3390/healthcare13121402 (PMC12193176; doi:10.3390/healthcare13121402)
Supplement: Supplementary file 1 [file healthcare-13-01402-s001.zip › healthcare-3625054-Table S1.pdf]

**Table S1.** All mean values from the assessment time points - Moments (Pre, D1 to D10, and Post) for both sequences A and B.

| Variable | Seq | Pre - Rest | | Day 1 - D1 | | Day 2 - D2 | | Day 3 - D3 | | Day 4 - D4 | | Day 5 - D5 | | Day 6 - D6 | | Day 7 - D7 | | Day 8 - D8 | | Day 9 - D9 | | Day 10 - D10 | | Post | |
|---|---|---|---|---|---|---|---|---|---|---|---|---|---|---|---|---|---|---|---|---|---|---|---|---|---|
| | | Mean | SD | Mean | SD | Mean | SD | Mean | SD | Mean | SD | Mean | SD | Mean | SD | Mean | SD | Mean | SD | Mean | SD | Mean | SD | Mean | SD |
| **Mean RR** | **A** | 705.5 | 91.8 | 667.2 | 76.0 | 662.0 | 68.6 | 695.1 | 99.7 | 686.8 | 101.9 | 696.3 | 75.7 | 678.6 | 113.6 | 699.9 | 130.6 | 667.4 | 68.1 | 681.3 | 83.5 | 677.2 | 117.8 | 753.1 | 101.8 |
| | **B** | 678.2 | 101.5 | 640.6 | 109.5 | 684.7 | 117.1 | 627.9 | 118.0 | 655.4 | 95.5 | 679.0 | 89.6 | 651.7 | 95.7 | 689.0 | 88.9 | 644.5 | 87.8 | 652.7 | 112.2 | 653.7 | 85.6 | 713.1 | 104.6 |
| **Mean HR** | **A** | 86.2 | 14.0 | 91.9 | 11.1 | 92.2 | 9.9 | 87.2 | 8.9 | 89.9 | 14.1 | 87.9 | 9.8 | 91.3 | 15.4 | 84.7 | 12.9 | 89.0 | 12.3 | 88.9 | 13.2 | 87.8 | 12.8 | 81.8 | 11.0 |
| | **B** | 90.9 | 13.5 | 96.4 | 15.4 | 90.3 | 14.5 | 94.9 | 14.6 | 93.7 | 12.8 | 90.2 | 11.6 | 92.6 | 10.8 | 89.1 | 11.5 | 92.0 | 10.0 | 95.0 | 15.0 | 91.1 | 14.3 | 86.8 | 12.9 |
| **SDNN** | **A** | 67.3 | 18.2 | 62.6 | 27.3 | 55.2 | 17.2 | 62.3 | 18.0 | 58.6 | 23.2 | 60.2 | 18.1 | 51.8 | 15.7 | 58.5 | 23.7 | 52.5 | 12.9 | 51.3 | 12.5 | 55.7 | 22.4 | 66.9 | 25.6 |
| | **B** | 44.9 | 13.8 | 38.4 | 16.9 | 42.1 | 20.1 | 37.8 | 16.7 | 43.5 | 21.5 | 49.3 | 20.3 | 48.9 | 22.2 | 48.4 | 20.3 | 44.2 | 16.3 | 49.8 | 25.1 | 53.7 | 25.9 | 52.6 | 20.5 |
| **RMSSD** | **A** | 39.2 | 19.5 | 31.9 | 13.3 | 32.6 | 10.7 | 30.7 | 13.4 | 35.1 | 18.7 | 36.8 | 18.0 | 32.9 | 16.4 | 37.5 | 24.4 | 30.4 | 10.3 | 35.3 | 17.0 | 36.1 | 23.7 | 50.4 | 27.9 |
| | **B** | 30.6 | 11.6 | 27.1 | 15.5 | 31.3 | 18.9 | 26.7 | 17.7 | 26.4 | 15.1 | 34.7 | 15.3 | 26.8 | 13.3 | 29.4 | 13.6 | 29.7 | 13.8 | 30.8 | 17.9 | 32.3 | 14.2 | 44.6 | 24.9 |
| **pNN50** | **A** | 19.0 | 16.5 | 13.2 | 10.5 | 11.4 | 8.5 | 10.0 | 7.5 | 14.0 | 13.7 | 14.8 | 14.3 | 14.6 | 12.1 | 18.5 | 18.2 | 10.2 | 6.5 | 13.5 | 11.5 | 15.4 | 16.6 | 25.7 | 21.8 |
| | **B** | 14.8 | 16.7 | 9.6 | 11.6 | 12.1 | 15.1 | 9.9 | 12.1 | 9.8 | 11.0 | 15.9 | 10.8 | 7.6 | 7.9 | 10.7 | 11.7 | 11.5 | 10.7 | 9.3 | 7.3 | 13.0 | 8.9 | 21.1 | 16.3 |
| **LF n.u.** | **A** | 67.2 | 15.7 | 76.7 | 10.7 | 75.2 | 11.8 | 77.9 | 11.3 | 74.0 | 14.9 | 74.5 | 9.6 | 74.9 | 10.7 | 77.0 | 11.2 | 76.5 | 11.1 | 71.4 | 14.2 | 77.9 | 15.3 | 65.9 | 10.7 |
| | **B** | 65.4 | 8.1 | 69.3 | 9.2 | 66.5 | 10.1 | 72.8 | 14.3 | 66.7 | 11.6 | 61.0 | 14.5 | 73.1 | 12.7 | 72.5 | 11.7 | 72.7 | 10.2 | 74.4 | 10.6 | 72.8 | 8.1 | 63.5 | 12.8 |
| **HF n.u.** | **A** | 32.6 | 10.3 | 23.2 | 10.7 | 24.7 | 11.8 | 22.1 | 11.3 | 25.9 | 14.8 | 25.5 | 9.6 | 25.0 | 10.7 | 22.8 | 11.2 | 23.4 | 11.1 | 28.3 | 14.1 | 22.0 | 15.3 | 33.8 | 10.6 |
| | **B** | 33.9 | 8.1 | 30.5 | 9.2 | 33.3 | 10.1 | 27.0 | 14.3 | 33.2 | 11.6 | 38.8 | 14.4 | 26.8 | 12.7 | 27.5 | 11.7 | 27.2 | 10.2 | 25.3 | 10.4 | 27.1 | 8.1 | 36.4 | 12.7 |
| **LF/HF** | **A** | 2.3 | 1.5 | 3.8 | 2.5 | 3.2 | 1.7 | 3.0 | 1.5 | 4.1 | 3.0 | 3.4 | 1.4 | 4.0 | 3.0 | 3.7 | 2.0 | 3.2 | 2.1 | 3.1 | 1.8 | 4.1 | 1.9 | 2.2 | 0.9 |
| | **B** | 2.1 | 0.7 | 2.7 | 1.5 | 2.1 | 1.3 | 3.1 | 1.9 | 1.9 | 0.8 | 2.1 | 1.7 | 3.1 | 1.6 | 3.4 | 2.1 | 3.2 | 1.5 | 3.1 | 2.1 | 2.5 | 0.8 | 1.6 | 0.8 |
| **SD1** | **A** | 27.8 | 13.8 | 22.6 | 9.4 | 23.1 | 7.5 | 21.7 | 9.4 | 24.9 | 13.2 | 26.1 | 12.8 | 23.3 | 11.6 | 26.5 | 17.3 | 21.5 | 7.3 | 25.0 | 12.1 | 25.6 | 16.8 | 35.7 | 19.8 |
| | **B** | 21.7 | 8.2 | 19.2 | 11.0 | 22.2 | 13.4 | 18.9 | 12.5 | 18.7 | 10.7 | 24.6 | 10.8 | 19.0 | 9.4 | 20.8 | 9.6 | 21.0 | 9.8 | 21.8 | 12.6 | 22.9 | 10.0 | 31.6 | 17.6 |
| **SD2** | **A** | 88.9 | 23.1 | 84.0 | 36.9 | 74.4 | 23.5 | 83.4 | 22.8 | 78.4 | 30.8 | 81.5 | 24.3 | 69.2 | 19.9 | 77.8 | 30.0 | 73.6 | 20.5 | 69.6 | 17.0 | 74.1 | 28.0 | 88.6 | 33.6 |
| | **B** | 59.5 | 18.1 | 50.7 | 21.8 | 60.4 | 32.6 | 49.8 | 20.5 | 57.0 | 27.0 | 63.7 | 24.7 | 64.9 | 28.1 | 64.8 | 28.2 | 58.7 | 21.3 | 65.1 | 30.4 | 70.3 | 32.4 | 68.6 | 26.5 |

Seq: Sequence; A: Participants first engaged with the abstract interaction via the webcam interface, followed by the concrete task using the touchscreen interface; B: Participants first completed the concrete interaction using the touchscreen interface, followed by the abstract task utilizing the webcam interface; SD: Standard Deviation.
