# Supplementary material for: Heart Rate Variability in Adolescents with Autistic Spectrum Disorder Practicing a Virtual Reality Using Two Different Interaction Devices (Concrete and Abstract): A Prospective Randomized Crossover Controlled Trial
Source: Healthcare (Basel). 2025 Jun 12;13(12):1402. doi: 10.3390/healthcare13121402 (PMC12193176; doi:10.3390/healthcare13121402)
Supplement: Supplementary file 1 [file healthcare-13-01402-s001.zip › healthcare-3625054-Table S2.pdf]

Table S2. Summary of MANOVA Results: F Values, p-Values, Eta partial square (η_p^2), and Observed Power for the Main Analyses. Only data with statistical significance were reported

**Variable**	**Sequence**	**Pre *versus* Post**			**Pre *versus* D1**			**Pre *versus* D10**			**D1 *versus* D5**		
		Moments	*Moments x Sequence*	*Sequence*	*Moments*	*Moments x Sequence*	*Sequence*	*Moments*	*Moments x Sequence*	*Sequence*	*Moments*	*Moments x Sequence*	*Sequence*
		F; p value; η_p^2; op (DF = 1, 20)			*F; p value; η_p^2; op (DF = 1, 20)*			*F; p value; η_p^2; op (DF = 1, 20)*			*F; p value; η_p^2; op (DF = 1, 20)*		
**Mean RR**	**A**<br>**B**	**4.06; 0.058; 0.17; 0.48**	-	-	-	-	-	-	-	-	-	-	-
**Mean HR**	**A**<br>**B**	-	-	-	-	-	-	-	-	-	-	-	-
**SDNN**	**A**<br>**B**	-	-	**6.36; 0.020; 0.24; 0.67**	-	**5.96; 0.024; 0.23; 0.64**	-	-	**4.87; 0.039; 0.19; 0.55**	-	-	-	-
**RMSSD**	**A**<br>**B**	**5.77; 0.026; 0.22; 0.63**	-	-	-	-	-	-	-	-	-	-	-
**pNN50**	**A**<br>**B**	-	-	-	-	-	-	-	-	-	-	-	-
**LF n.u.**	**A**<br>**B**	-	-	-	-	**7.23; 0.014; 0.27; 0.72**	-	**14.02; 0.001; 0.41; 0.94**	-	-	-	-	-
**HF n.u.**	**A**<br>**B**	-	-	-	-	**6.12; 0.022; 0.23; 0.65**	**4.51; 0.046; 0.18; 0.52**	**10.13; 0.005; 0.33; 0.85**	-	-	-	-	-
**LF/HF**	**A**<br>**B**	-	-	-	**8.14; 0.010; 0.28; 0.77**	**8.24; 0.009; 0.29; 0.78**	-	**18.15; <0.001; 0.47; 0.98**	**7.74; 0.011; 0.28; 0.75**	-	-	-	-
**SD1**	**A**<br>**B**	**5.78; 0.026; 0.22; 0.63**	-	-	-	-	-	-	-	-	-	-	-
**SD2**	**A**<br>**B**	-	-	**6.74; 0.017; 0.25; 0.69**	-	**5.46; 0.030; 0.21; 0.60**	**4.58; 0.045; 0.18; 0.53**	-	**4.67; 0.043; 0.18; 0.54**	-	-	-	-

A: Participants first engaged with the abstract interaction via the webcam interface, followed by the concrete task using the touchscreen interface; B: Participants first completed the concrete interaction using the touchscreen interface, followed by the abstract task utilizing the webcam interface; Moments of assessments (Pre, D1 to D10, and Post); DF: Degrees of freedom.
